# Methyl Carbonium Ion Migration during the Reaction of 4-Chloro-5-methoxyl-3(*2H*)-pyridazinone with Trifluoroethylation Agents

**DOI:** 10.3390/molecules14020777

**Published:** 2009-02-13

**Authors:** Qin Li, Guichun Lin, Li Liu, Zhenjun Yang, Li-He Zhang

**Affiliations:** The State Key Laboratory of Natural and Biomimetic Drugs, School of Pharmaceutical Sciences, Peking University, Xue Yuan Road ^#^38, Beijing 100191, P.R. China; E-mail: liqin@bjmu.edu.cn (Q.L.)

**Keywords:** 4,5-Dichloro-3(*2H*)-pyridazinone, 4-Chloro-5-methoxy-3(2*H*)-pyridazinone, β-trifluoroethylation, Oxonium, Methyl group migration, Alkylation.

## Abstract

To synthesize 4-chloro-5-methoxy-2-(β-trifluoroethyl)-3(2*H*)-pyridazinone (**4**), the reactions of 4-chloro-5-methoxy-3(2*H*)-pyridazinone (**5**) with RCH_2_CF_3_ (R = I, TsO, MsO, TfO) in different solvents were studied. It was found that methyl group migration took place during this reaction. An oxonium salt **9** was suggested as the active intermediate for the formation of the byproduct 4-chloro-5-methoxy-2-methyl-3(2*H*)-pyridazinone (**7**) and 4-chloro-2-methyl-5-(β-Trifluoroethoxy)-3(2)-pyridazinone (**8**).

## Introduction

**Scheme 1:**
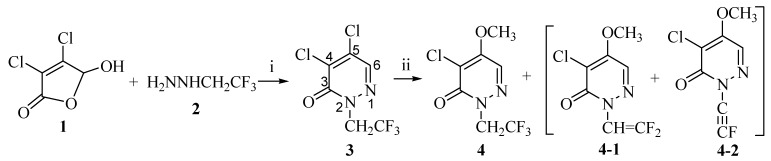
Direct introduction of trifluoroethyl group to the diazine 3.

It is well-known that the incorporation of fluorine atoms into organic molecules often has profound effects on their chemical and physical properties [1,2], thus there has been considerable interest in organofluorine compounds as pharmaceutical and agrochemical agents [3,4]. Among fluorine- containing groups, the β-trifluoroethyl moiety has been found in many drug molecules, where it can retard or prevent oxidative dealkylation of an *N*-, *S*-, or *O*-alkyl function [5,6,7,8,9,10]. 2-Substituted 4-chloro-5-methoxyl-3(*2H*)-pyridazinones are key intermediates in the synthesis of agrochemically and pharmaceutically important 2,4,5-trisubstituted-3(2*H*)-pyridazinones [11,12,13,14,15]. Although several approaches to nonfluorinated 2-substituted 4-chloro-5-methoxy-3(2*H*)-pyridazinones have been well documented in the prior literatures [16,17], β-trifluoroethylated 2,4,5-trisubstituted-3(2*H*)-pyridazinones are much less known. This is ascribed to the absence of practical and convenient methods for the introduction of the β-trifluoroethyl group into organic compounds. We failed to synthesize 4-chloro-5-methoxy-2-(β-trifluoroethyl)-3(2*H*)-pyridazinone (**4**) by treatment of 4,5-dichloro-2-(β-trifluoroethyl)-3(2*H*)-pyridazinone (**3**), which was prepared from reaction of mucochloric acid (**1**) and β-trifluoroethyl hydrazine (**2**) [18], with sodium methoxide (MeONa) in methanol under reflux, because the β-trifluoroethyl group was sensitive to the strong base. Elimination happened after treating **3** with strong base, and instead of compound **4**, a low yield of a mixture (most probably a mixture of **4-1** and **4-2**, according ^1^H-NMR) was obtained, which could contain trace amounts of **4** [19]. On the other hand, a weaker base like potassium carbonate (K_2_CO_3_) failed to catalyze the substitution (Scheme 1). In this paper, we report the β-trifluoroethylation of 4-chloro-5-methoxy-3(2*H*)-pyridazinone (**5**) to synthesize compound **4**, while proposing formation of an oxonium salt **9** during the reaction of compound **5** with LCH_2_CF_3_ (L = I, TsO, MsO, TfO ) under different conditions, to further form 4-chloro-5-methoxy-2-methyl-3(2H)-pyridazinone (**7**) and 4-chloro-2-methyl-5-trifluoroethyl-3(2H)-pyridazinone (**8**), in different ratios and yields, respectively.

*Reagents and conditions*: i). C_2_H_5_OH, reflux; ii). K_2_CO_3_/CH_3_OH, reflux or CH_3_ONa/CH_3_OH, reflux.

## Results and Discussion

4,5-Dichloro-2-(β-trifluoroethyl)-3(*2H*)-pyridazinone (**3**) can be prepared from the reaction of mucochloric acid (**1**) and β-trifluoroethyl hydrazine (**2**) [18]. However, the methoxyl substitution on the 5-chloro of compound **3** was very difficult due to the elimination of β-trifluoroethyl group by the base, resulting in a mixture of compound **4**, **4-1** and **4-2** [17,18,19] (Scheme 1). Therefore, we tried to prepare **4** by β-trifluoroethylation of the corresponding 4-chloro-5-methoxy-3(*2H*)-pyridazinone (**5**) with several different agents.

Reaction of 5 with β-trifluoroethyl methanesulfonate (MsOCH_2_CF_3_) in the presence of K_2_CO_3_ in HMPA at 140 ^o^C (Table 1, entry 5) afforded exclusively 4-chloro-2-methyl-5-(β-trifluoroethyl)-3(2H)-pyridazinone (**8**) in 65% yield. When the reaction was carried out in DMF, we obtained only 4-chloro-5-methoxy-2-methyl-3(2H)-pyridazinone (7) in 40% yield. When sodium hydride (NaH) was used instead of K_2_CO_3 _(Table 1, entry 6), a mixture of compound **4** and **8** was obtained.

**Scheme 2:**
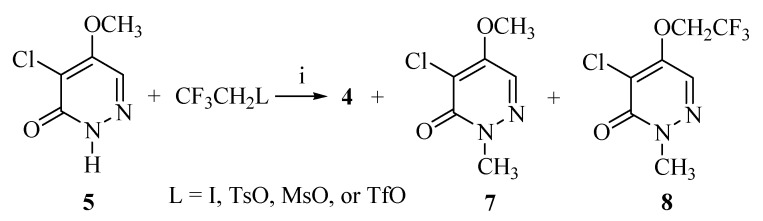
Synthesis of compound **4** with **7** and **8** as by-products.

*Reagents and conditions*: i). see Table 1.

**Table 1:** Reactions of compound **5** withdifferent alkylation reagents.

Entry		Condition	Product (yield)	Time
1	ICH_2_CF_3_	K_2_CO_3_/DMF, 80 °C	**4** (5%), **7** (20%)	12 h
2	TsOCH_2_CF_3_	K_2_CO_3_/DMF, 120 °C	No product	20 h
3	MsOCH_2_CF_3_	K_2_CO_3_/18-crown-6/DMF, 120 °C	**7** (40%)	20 h
4	MsOCH_2_CF_3_	K_2_CO_3_/18-crown-6/1,4-Dioxane, reflux	**4** (trace), **7** (35%)	20 h
5	MsOCH_2_CF_3_	K_2_CO_3_/18-crown-6/HMPA, 140 °C	**8** (45%)	20 h
6	MsOCH_2_CF_3_	NaH/18-crown-6/HMPA, 140 °C	**4** (15%), **8** (25%)	20 h
7	TfOCH_2_CF_3_	K_2_CO_3_/18-crown-6/HMPA, 60 °C	**4** (40%), **7** (25%)	24 h

**Figure 1:**
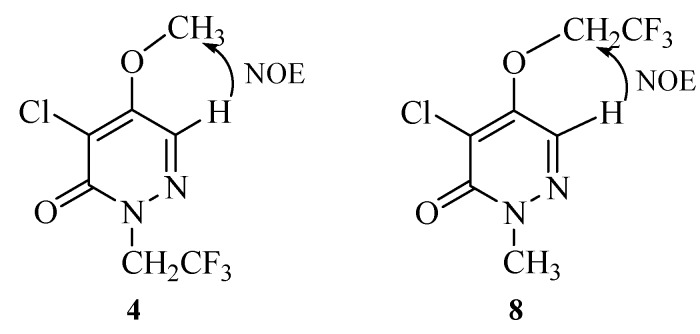
NOE between H-6 and methyl or trifluoroethyl group in compound **4** or **8**.

Compounds **4** and **8** are a pair of isomers. The substitution position on **4** and **8** was confirmed by 1D NOE NMR spectroscopy. Both **4** and **8** were subjected to a 1D NOE difference experiment. Irradiation of the methyl peak of **4** gave signal enhancement for protons at position 6, whereas, irradiation of the H-6 proton of **8** resulted in enhancement of the methylene proton NMR signal, in good agreement with the structural assignment (Figure 1).

Both compounds **7** and **8** have methyl groups on the *N*-2 position. To determine whether these methyl groups come from the solvents or from the substrate **5**, compound **5** was treated with β-trifluoroethyl methanesulfonate (MsOCH_2_CF_3_) in 1,4-dioxane, yielding **7** (35%) as the major product, with only trace amounts of **4**(Table 1, entry 4). It is obvious that compound **7** and **8** might be formed by the methyl migration from *O*-5 of compound **5**. When **5** was allowed to react with β-trifluoroethyl trifluoromethanesulfonate (TfOCH_2_CF_3_) in the presence of K_2_CO_3_ and 18-crown-6 in HMPA at 60 °C (Table 1, entry 7), compound **4** was obtained in 40% yield, while **7** was obtained in 25% yield. Reaction of **5** with β-trifluoroethyl iodide (CF_3_CH_2_I) in the presence of K_2_CO_3 _in DMF (entry 2) provided compound **4** in very low yield (5%), while **7** in 20% yield. We presumed that intramolecular elimination happened for CF_3_CH_2_I at basic condition and high temperature.

**Scheme 3:**
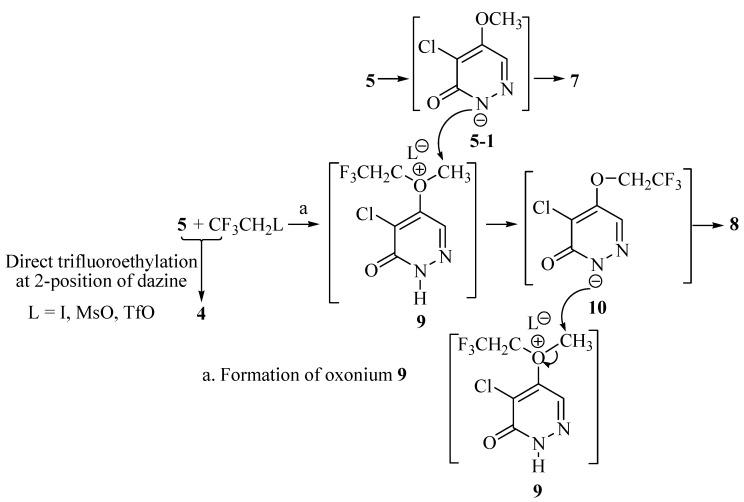
Possible mechanism for the formation of compounds **7**, **8** and oxonium ion **9**.

Based on these observations, a possible mechanism for the formation of compounds **4**, **7** and **8** was proposed (Scheme 3). ICH_2_CF_3 _andMsOCH_2_CF_3 _as alkylating reagents could attack *N*-2 of compound **5** to yield compound **4**, while the carbonium ion CF_3_CH_2_^+^could form the oxonium salt **9** with compound **5**. The formation ofcompound **8** was proposed by methyl carbonium ion migration from oxonium salt **9** to the intermediate **10**.Andoxonium salt **9** reactedwith compound **5** *via* an intermolecular methyl carbonium ion migration to yield compound **7 [20]**.

**Scheme 4:**
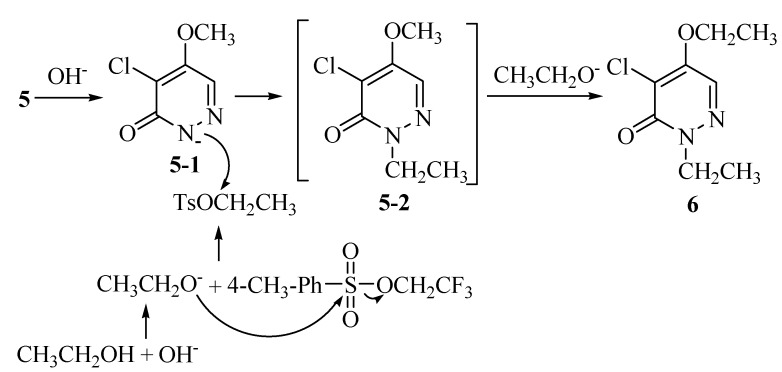
Ethylation of diazinone 5 at the 2 and 4-positions.

**Scheme 5:**
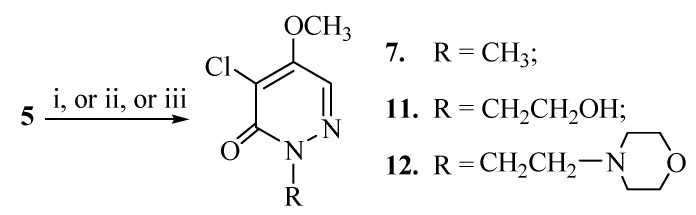
Alkylation at the 2-postion of diazinone 5.

We had investigated the reaction of compound **5** with trifluoroethyl p-toluenesulfonate (TsOCH_2_CF_3_) in the presence of sodium hydroxide (NaOH) in aqueous ethanol, which afforded 4-chloro-5-ethoxy-2-ethyl-3(2*H*)-pyridazinone (**6**) in 70% yield. The mechanism of formation of compound **6** as shown in Scheme 4 is suggested. The more basic ethoxyl group could competitively substitute the trifluoroethoxyl group to form TsOCH_2_CH_3_,which then reacted with **5**, leading to the *N*-2 ethylation of **5**. There placement of 5**-**methoxyl group on the formed *N*-2 ethylation intermediate gave **6**. We also investigated the reaction of compound **5** with other three alkylating reagents (Scheme 5). Methylation of compound **5** with iodomethane(CH_3_I) and potassium carbonate in DMF gave compound **7** in excellent yield.

Hydroxyethylation of **5** with sodium hydroxide and 2-bromoethanol also afforded the corresponding 4-chloro-2-(β-hydroxyethyl)-5-methoxy-3(2*H*)-pyridazinone (**11**) in good yield. Reaction of **5** with 1-chloro-2-(*N*-morpholino)ethane in the presence of potassium carbonate yielded the expected 4-chloro-5-methoxy-2-{β-(*N*-morpholino)}ethyl-3(2*H*)-pyridazinone (**12**). No carbonium ion shift product was detected in reaction of **5** with 2-bromoethanol or 2-(*N*-morpholino)-1-chloroethane.

*Reagents and conditions*: i). K_2_CO_3_/ CH_3_I/DMF, 50-60 °C, 2h; ii). 2N NaOH/BrCH_2_CH_2_OH/ CH_3_CH_2_OH, reflux, 3h; iii). K_2_CO_3_/ 2-(*N*-morpholino)-1-chloroethane.

## Conclusions

In summary, the reaction of 4-chloro-5-methoxy-3(2*H*)-pyridazinone (**5**) with MsOCH_2_CF_3_ or TfOCH_2_CF_3_ in polar aprotic solvents lead to the formation of an oxonium salt **9** which generated β-trifluoroethyl and methyl carbonium ions.Compound **8** was formed by methyl carbonium ion migration from *O*-5 of the intermediate **9** to *N*-2 of intermediate **10**.Oxonium salt **9** reacted with compound **5** *via* an intermolecular methyl carbonium ion migration, yielding compound **7**. The β-trifluoroethyl carbonium ion could also be an alkylating reagent, reacting directly with compound **5** to furnish 4-chloro-5-methoxy-2-(β-trifluoroethyl)-3(2H)-pyridazinone (**4**).

## Experimental

### General

Column chromatography was performed on silica gel (200-300 mesh). Melting points were determined on a Mel-temp II and are uncorrected. NMR spectra were recorded on a Varian INOVA-500 NMR spectrometer operating at 499.821 MHz for ^1^H and 125.71 MHz for ^13^C. Chemical shifts (d) are referenced to internal TMS and reported in ppm. Splitting patterns were as follows: *s*, singlet; *br*, broad; *δ*, doublet; *t*, triplet; *q*, quartet; *m*, multiplet. Mass spectra were recorded under fast bombardment (FAB), on a Micromass Autospect high-resolution mass spectrometer unless noted. Elemental analyses were recorded on PE-240C. Infrared spectra (IR) were obtained with a Nicolet NEXUS-470 FTIR spectrometer as KBr pellets.

*4,5-Dichloro-2-(β-trifluoroethyl)-3(2H)-pyridazinone* (**3**). Mp 83-85 °C. MS (ESI): *m/z* 247 [M+1]^+^. Anal. calcd. for C_6_H_3_Cl_2_ F_3_N_2_O: C 29.18, H 1.22, N 11.34; Found: C 29.19, H 1.26, N 11.34.

*4-Chloro-5-methoxy-3(2H)-pyridazinone* (**5**).Mp 123-124 ^o^C.^ 1^H-NMR (DMSO-d_6_) *δ* 11.59 (br, 1 H, H-2), 7.78 (s, 1 H, H-6), 4.063 (s, 3 H, -OCH_3_).

*4-Chloro-5-ethoxy-2-ethyl-3(2H)-pyridazinone* (**6**): To a solution of **5** (1.0 g, 6.2 mmol) in C_2_H_5_OH (60 mL) were added TsOCH_2_CF_3_ (6.2 g, 25.2 mmol) and 2 N NaOH (10 mL). The mixture was stirred under reflux for 14 h. After cooling to room temperature, the reaction mixture was evaporated to dryness under reduced pressure. The residue was dissolved in CH_2_Cl_2_ and solution was washed with brine. The organic layer was separated, dried over anhydrous sodium sulfate (Na_2_SO_4_) and concentrated. The resulting residue was purified by column chromatography (hexane/ethyl acetate) to give compound **6** (877 mg, 70%) as a colorless solid. Mp 64-66 °C;^ 1^H-NMR (CDCl_3_) *δ* 7.79 (s, 1 H, H-6), 4.32 (q, *J* = 7.0 Hz, 2 H, OCH_2_CH_3_), 4.24 (q, 2H, *J* = 7.0 Hz, N-CH_2_CH_3_), 1.50 (t, *J* = 7.0 Hz, 3 H, -OCH_2_CH_3_), 1.37 (t, *J* = 7.0Hz, 3 H, N-CH_2_CH_3_); ^13^C NMR (CDCl_3_) *δ* 158.3 (C=O), 154.3 (C-6), 126.7 (C-5), 116.5 (C-4), 66.4 (OCH_2_CH_3_), 47.6 (N-CH_2_CH_3_), 14.8 (-OCH_2_CH_3_), 13.4 (-NCH_2_CH_3_); MS (FAB): *m/z* 203 [M+1]^+^; Anal. calcd. for C_8_H_11_Cl_1_N_2_O_2_: C 47.42, H 5.47, N 13.82; Found: C 47.13, H 5.44, N, 13.48. 

### General procedure for the synthesis of compound **4**

To the solution of **5** in solvent were added LCH_2_CF_3_ and base. The mixture was stirred under reflux for 14 h. After cooling to room temperature, the reaction mixture was evaporated to dryness under reduced pressure. CH_2_Cl_2_ and water were added. The organic layer was separated and dried over anhydrous sodium sulfate (Na_2_SO_4_). After evaporation, the residue was purified by column chromatography (hexane/ethylacetate) to give compound **4** and **7** or **8**.

*4-Chloro-5-methoxy-2-(β-Trifluoro)ethyl-3(2H)-pyridazinone* (**4**). White powder; mp 90-92 °C; IRn_max _(cm^--1^): 3318-2853 (m), 1671, 1609, 1466, 1385, 1320, 1286, 1267, 1219, 1150, 1106, 892, 772; ^1^H-NMR (CDCl_3_) *δ* 7.88 (s, 1 H, H-6), 4.82 (q, *J* = 8.5 Hz, 2 H, -NCH_2_-), 4.11 (s, 3 H, OCH_3_); ^13^C-NMR (CDCl_3_) *δ* 158.5 (C=O), 155.0 (C-6), 127.9 (C-5), 123.0 (q, ^1^*J*_C,F_ = 153.6 Hz, -CF_3_), 116.5 (C-4), 58.5 (-OCH_3_), 52.5 (q, ^2^*J*_C,F_ = 35.1 Hz, -N-CH_2_-); MS (EI): *m/z* 242.0 [M]^+^; Anal. calcd. for C_7_H_6_Cl_1_F_3_N_2_O_2_: C 34.66, H 2.49, N 11.55; Found: C 34.69, H 2.52, N 11.41.

*4-Chloro-5-methoxy-2-methyl-3(2H)-pyridazinone* (**7**). White powder; yield 75%; mp 131 - 133 ^o^C; ^1^H-NMR (CDCl_3_) *δ* 7.78 (s, 1 H, H-6), 4.07 (s, 3H, -OCH_3_), 3.83 (s, 3 H, N-CH_3_).

*4-Chloro-2-methyl-5-(β-Trifluoroethoxy)-3(2)-pyridazinone* (**8**). White powder. Mp164-166 °C. IRn_max_ (cm^-1^) 3409 - 2927 (m), 1653, 1602, 1468, 1397, 1334, 1301, 1271, 1208, 1170, 1111, 1000, 967, 876; ^1^H-NMR (CDCl_3_) *δ* 7.71 (s, 1 H, H-6), 4.60 (q, 2H,^ 3^*J*_H,F_ = 7.5 Hz, -OCH_2_-), 3.83 (s, 3 H, N-CH_3_); ^13^C- NMR (CDCl_3_) *δ* 158.5 (C=O), 153.5 (C-6), 127.9 (C-5), 122.3 (q, ^2^*J*_C,F_ = 153.6 Hz, -CF_3_), 120.1 (C-4), 67.6 (q,^ 2^*J*_C,F_ = 35.1 Hz, N-CH_2_-), 41.0 (-NCH_3_); MS (EI): *m/z* 242.0 [M]^+^; Anal. calcd. for C_7_H_6_Cl_1_F_3_N_2_O_2_: C 34.66, H 2.49, N 11.55; Found: C 34.76, H 2.56, N 11.38.

*4-Chloro-2-(β-hydroxyethyl)-5-methoxy-3(2H)-pyridazinone* (**11**). Mp: 166-168 °C; ^1^H-NMR (DMSO-d6): d 8.26 (s, 1 H, H-6), 4.83 (brs, 1 H, OH), 4.15 (t, *J* = 6.6 Hz, 2H, -NCH_2_-), 4.07 (s, 3 H, -OCH_3_), 3.69 (t, *J* = 4.5 Hz, 2 H, -OCH_2_); MS (FAB): *m/z* 205.0 [M+1]^+^; Anal. calcd. for C_7_H_9_Cl_1_N_2_O_3_: C 41.09, H 4.43, N 13.69; Found: C 40.80, H 4.46, N 13.74.

*4-Chloro-5-methoxy-2-(β-N-morphilinoethyl)-3(2H)-pyridazinone* (**12**). Mp 170-172 °C. ^1^H NMR (DMSO-d_6_): *δ* 8.24 (s, 1 H, H-3), 4.21 (t, *J* = 6.6 Hz, 2H, -NCH_2_-), 4.06 (s, 3 H, -OCH_3_), 4.08 (t, *J* = 4.5 Hz, 4 H, 2 x OCH_2_), 2.62 (t, *J* = 6.6 Hz, 2 H, -NCH_2_), 2.39 (t, *J* = 4.5 Hz, 4 H, 2 x -NCH_2_). MS (EI): *m/z* 273 [M]^+^. Anal. Calcd. for C_11_H_16_Cl_1_N_3_O_3_: C 48.27, H 5.89, N 15.35; Found: C 48.50, H 6.02, N 15.52.

## References

[1] Kirsch P. (2004). Modern Fluoroorganic Chemistry.

[2] Chambers R.D. (2004). Fluorine in Organic Chemistry.

[3] Ismail F.M.D. (2002). Important fluorinated drugs in experimental and clinical use. J. Fluorine Chem..

[4] Jeschke P. (2004). The unique role of fluorine in the design of active ingredients for modern crop protection. ChemBioChem.

[5] Quazepam (1996). Merck Index.

[6] Halazepamin (1996). Merck Index.

[7] Steinmann M., Topliss J.G., Alekel R., Wong Y.-S., York E.E. (1973). 1-Poly(fluoroalkyl)benzo- diazepines. J. Med. Chem..

[8] Flecainide (1996). Merck Index.

[9] Banitt E.H., Schmid J.R., Mendel A. (1975). Antiarrhythmics. N-(Aminoalkylene)trifluoroethoxy-benzamides and N-(aminoalkylene)trifluoroethoxynaphthamides. J. Med. Chem..

[10] Banitt E.H., Bronn W.R., Coyne W.E., Schmid J.R. (1977). Antiarrhythmics. 2. Synthesis and antiarrhythmic activity of N-(piperidylalkyl)trifluoroethoxybenzamides. J. Med. Chem..

[11] Maes B.U.W., R’kyek O., Košmrlj J., Lemière G.L.F., Esmans E., Rozenski J., Dommisse R.A., Haemers A. (2001). Suzuki reactions on chloropyridazinones: an easy approach towards arylated 3(2*H*)-pyridazinones. Tetrahedron.

[12] Riedl Z., Maes B.U.W., Monsieurs K., Lemiere G.L.F., Matyus P., Hajos G. (2002). Synthesis of new pyridazino[4,5-*c*]isoquinolinones by Suzuki cross-coupling reaction. Tetrahedron.

[13] Frank H., Heinisch G., Ellis G.P., West G.B. (1990). Progress in Medicinal Chemistry.

[14] Rkyek O., Maes B.U.W., Jonckers T.H.M., Lemiere G.L.F., Dommisse R. A. (2001). New enediyne derivatives: synthesis of symmetrically and unsymmetrically disubstituted 4,5-dialkynyl-3(2*H*)-pyridazinones. Tetrahedron.

[15] Dury K. (1965). New methods in the chemistry pyridazones. Angew. Chem. Int. Ed. Engl..

[16] Lyga J.W. (1988). The reaction of 2-substituted-4,5-dihalo-3(*2H*)-pyridazinones with alkoxides and alkylthiolates. J. Heterocyclic Chem..

[17] Cho S-D, Choi W-Y, Yoon Y.-J. (1996). Concurrent alkylation-methoxylation of 4,5-dihalopyridazin-6-ones and synthesis of 5-halo-4-hydroxypyridazin-6-ones. J. Heterocyclic Chem..

[18] David T.M. (1953). Mucochloric acid. II. Reactions of the aldehyde group. J. Am. Chem. Soc..

[19] Hine J., Ghirardelli R.G. (1958). The SN- reactivity of β-fluoroethyl iodides. J. Org. Chem..

[20] Quinn K.J., Biddick N.A., De Christopher B. A. (2006). Ring expansion of *trans*-divinyl ethylene oxide by oxonium ylide [2,3] sigmatropic rearrangement. Tetrahedron Lett..

